# A novel mechanism for acute colonic pseudo‐obstruction revealed by high‐resolution manometry: A case report

**DOI:** 10.14814/phy2.14950

**Published:** 2021-07-07

**Authors:** Cameron I. Wells, Nira Paskaranandavadivel, Peng Du, James A. Penfold, Armen Gharibans, Ian P. Bissett, Greg O'Grady

**Affiliations:** ^1^ Department of Surgery Faculty of Medical and Health Sciences The University of Auckland Auckland New Zealand; ^2^ Auckland Bioengineering Institute The University of Auckland Auckland New Zealand

**Keywords:** acute colonic pseudo‐obstruction, case report, colonic manometry, colonic motility, Ogilvie's syndrome, rectosigmoid brake

## Abstract

**Background:**

Acute colonic pseudo‐obstruction (ACPO) is a severe form of colonic dysmotility and is associated with considerable morbidity. The pathophysiology of ACPO is considered to be multifactorial but has not been clarified. Although colonic motility is commonly assumed to be hypoactive, there is little direct pathophysiological evidence to support this claim.

**Methods:**

A 56‐year‐old woman who developed ACPO following spinal surgery underwent 24 h of continuous high‐resolution colonic manometry (1 cm resolution over 36 cm) following endoscopic decompression. Manometry data were analyzed and correlated with a three‐dimensional colonic model developed from computed tomography (CT) imaging.

**Results:**

The distal colon was found to be profoundly hyperactive, showing near‐continuous non‐propagating motor activity. Dominant frequencies at 2–6 and 8–12 cycles per minute were observed. The activity was often dissociated and out‐of‐phase across adjacent regions. The mean amplitude of motor activity was higher than that reported from pre‐ and post‐prandial healthy controls. Correlation with CT imaging suggested that these disordered hyperactive motility sequences might act as a functional pseudo‐obstruction in the distal colon resulting in secondary proximal dilatation.

**Conclusions:**

This is the first detailed description of motility patterns in ACPO and suggests a novel underlying disease mechanism, warranting further investigation and identification of potential therapeutic targets.

## Graphical abstract

The distal colon is commonly assumed to be hypoactive in acute colonic pseudo‐obstruction (ACPO), though there are no previously‐published data supporting this claim. We present the first‐ever recordings of colonic motility in ACPO, demonstrating the distal colon was markedly hyperactive with disorganised, non‐propagating motor activity. These contractions may act as a functional obstruction, leading to pseudo‐obstruction and proximal colonic dilatation.

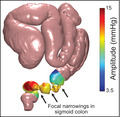

## 1 INTRODUCTION

Acute colonic pseudo‐obstruction (ACPO) is a severe form of colonic dysmotility resulting in functional obstruction of the large bowel, with ischemia or perforation occurring in up to 15% of patients (Nanni et al., 1982). The pathophysiology of ACPO is considered to be multifactorial and likely results from an imbalance in autonomic signaling to the colon; however, the precise abnormalities in colonic motility have not been defined (Wells et al., 2017). Sir William Ogilvie first described the condition in 1948 and proposed it was due to “sympathetic deprivation” of the distal colon (Ogilvie, 1948), though modern theories implicate overactive sympathetic or reduced parasympathetic activity (Wells et al., 2017). The distal colon is commonly assumed to be hypoactive in ACPO (Pereira et al., 2015; Saunders & Kimmey, 2005); however, no published physiological data support this claim and the aberrations of colonic motility resulting in ACPO have never been characterized (Wells et al., 2017). This report describes a novel pathophysiological mechanism of ACPO in a patient following spinal surgery, reported according to the CARE guidelines (Gagnier et al., 2013).

## 2 CASE REPORT

A 56‐year‐old woman underwent an elective right L5 nerve root decompression for L5/S1 foraminal stenosis. Prior to the operation, she had a stable daily bowel habit. Her past medical history included a previous episode of ACPO following a redo lumbar spinal fusion approximately 1 year prior, depression for which she took mirtazapine regularly, and an intensive care unit admission related to *Clostridium difficile* infection and dilated cardiomyopathy following a posterior lumbar fusion 6 years earlier.

**FIGURE 1:**
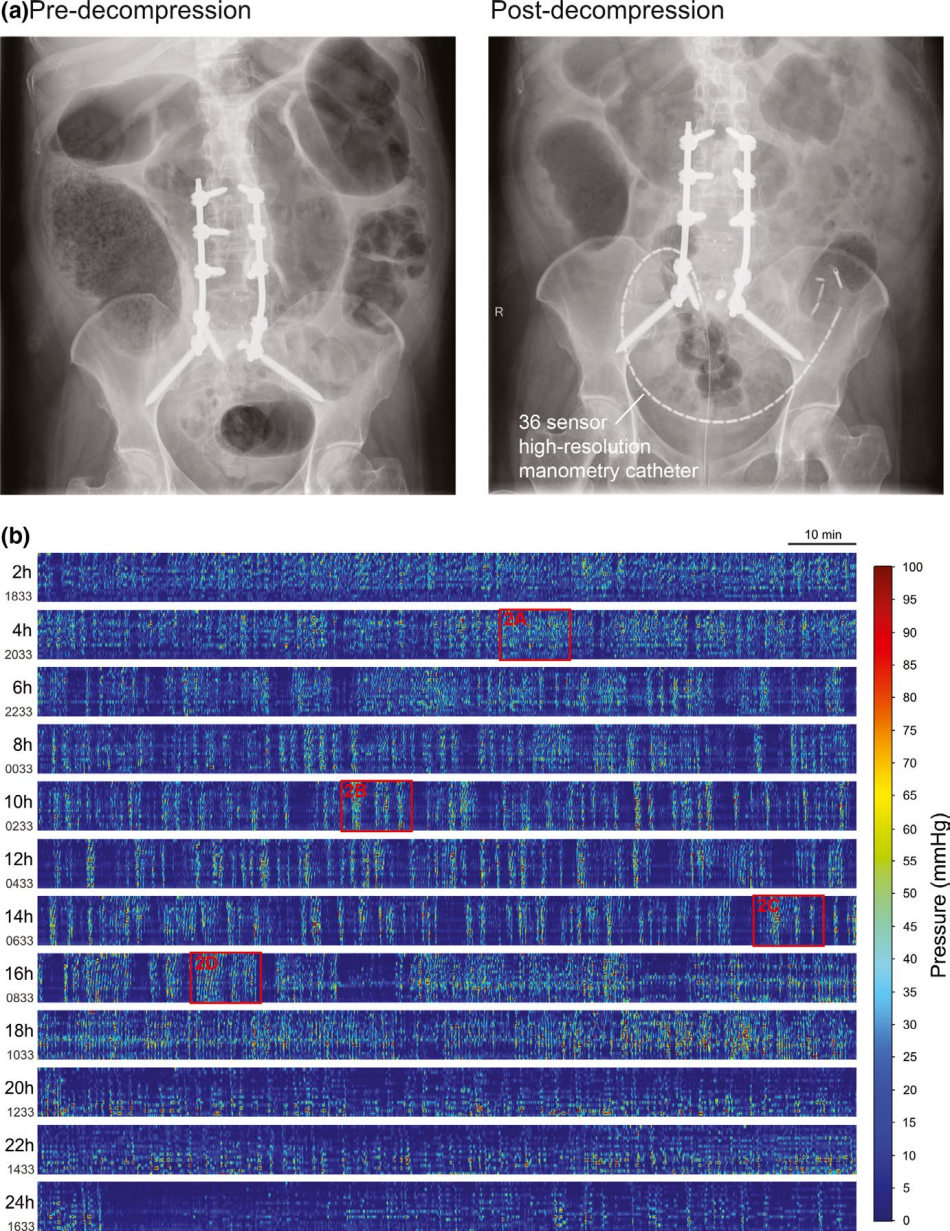
(a) Abdominal radiographs prior to and following endoscopic decompression and insertion of high‐resolution manometry catheter. (b) Overview of the entire 24 h recording period showing marked distal colonic hyperactivity. Each horizontal bar represents a 2 h period. The time of day at the start of each 2 h block is shown on the left of the figure. Red boxes represent periods of data displayed in Figure 2

The operation was completed under general anesthesia without an epidural. There were no intraoperative complications. Patient‐controlled intravenous oxycodone was used for initial postoperative analgesia and this was changed to oral oxycodone on the morning of the postoperative day (POD) 1. On POD 3, she developed abdominal pain and distension. She had not passed a bowel motion since prior to the operation. Abdominal radiograph and computed tomography (CT) showed ACPO and caecal dilatation to 9.8 cm, without the evidence of mechanical obstruction, ischemia, or perforation (Figure 1a, Video S1). Serum electrolyte concentrations (sodium, chloride, potassium, calcium, magnesium, and phosphate) were all within normal ranges. She proceeded for colonoscopic decompression on POD 4, which revealed a dilated colon with normal mucosa. Following decompression, a 36‐sensor fiber‐optic high‐resolution (HR) manometry catheter was endoscopically inserted in the distal colon, as previously described (Figure 1a) (Vather et al., 2018). A flatus tube was not inserted.

Continuous HR manometric recordings were obtained for 24 h, after which the catheter was removed. During the recording period, the patient's abdominal pain had improved and she had no oral intake other than small amounts of clear fluids. She passed flatus on the morning following catheter insertion, but did not pass any further flatus or stool during the rest of the recordings. She received 10 mg of oral oxycodone during the first hour following catheter insertion and a further 5 mg dose at 11:50 a.m. the following morning. Recurrent symptoms on POD 6 required a further endoscopic decompression and a second insertion of the manometry catheter was not performed. The patient recovered well and was discharged on POD 11 without further intervention. Neostigmine was not given at any stage during the hospital admission.

## 3 HIGH‐RESOLUTION COLONIC MANOMETRY

### 3.1 Methods

HR manometry data were recorded using a 36‐sensor fiber‐optic manometry catheter with 1 cm spacing between sensors (Arkwright Technologies). The catheter was attached to a spectral interrogator unit (FBG‐scan 804D; FBGS International) and pressure events were recorded using a LabVIEW© interface (National Instruments). Colonic manometry signals were filtered as previously described: baseline drift was removed using an envelope detection technique, followed by the removal of synchronous events and high‐frequency noise (Paskaranandavadivel et al., 2021). The mean amplitude over 15 min periods for every channel was also computed and averaged across all sensors. The dominant frequency of each sensor was measured by fast Fourier transform (FFT) with a window of 1 min and a shifting window of 48 s over the recorded period. Frequency detection was performed on signals that were filtered using a sixth‐order Butterworth lowpass filter with a cut‐off of 1 Hz/60 cpm. Dominant frequencies of all the sensors over each window were then categorized into discrete bands between 2 and 12 cpm. The percentage of each band within the total number of windows over the recorded period was calculated.

A three‐dimensional (3D) model of the patient's colon was constructed by the manual segmentation of the pre‐decompression abdominal CT scan in 3DSlicer 4.10.0 (http://www.slicer.org). To visualize HR manometry data with anatomical correlation, the mean amplitude and dominant frequency for the entire 24 h period were mapped to the center‐point of this model, along the region of the colon corresponding to the position of the HR manometry catheter (Davidson et al., 2011).

### 3.2 Results

**FIGURE 2:**
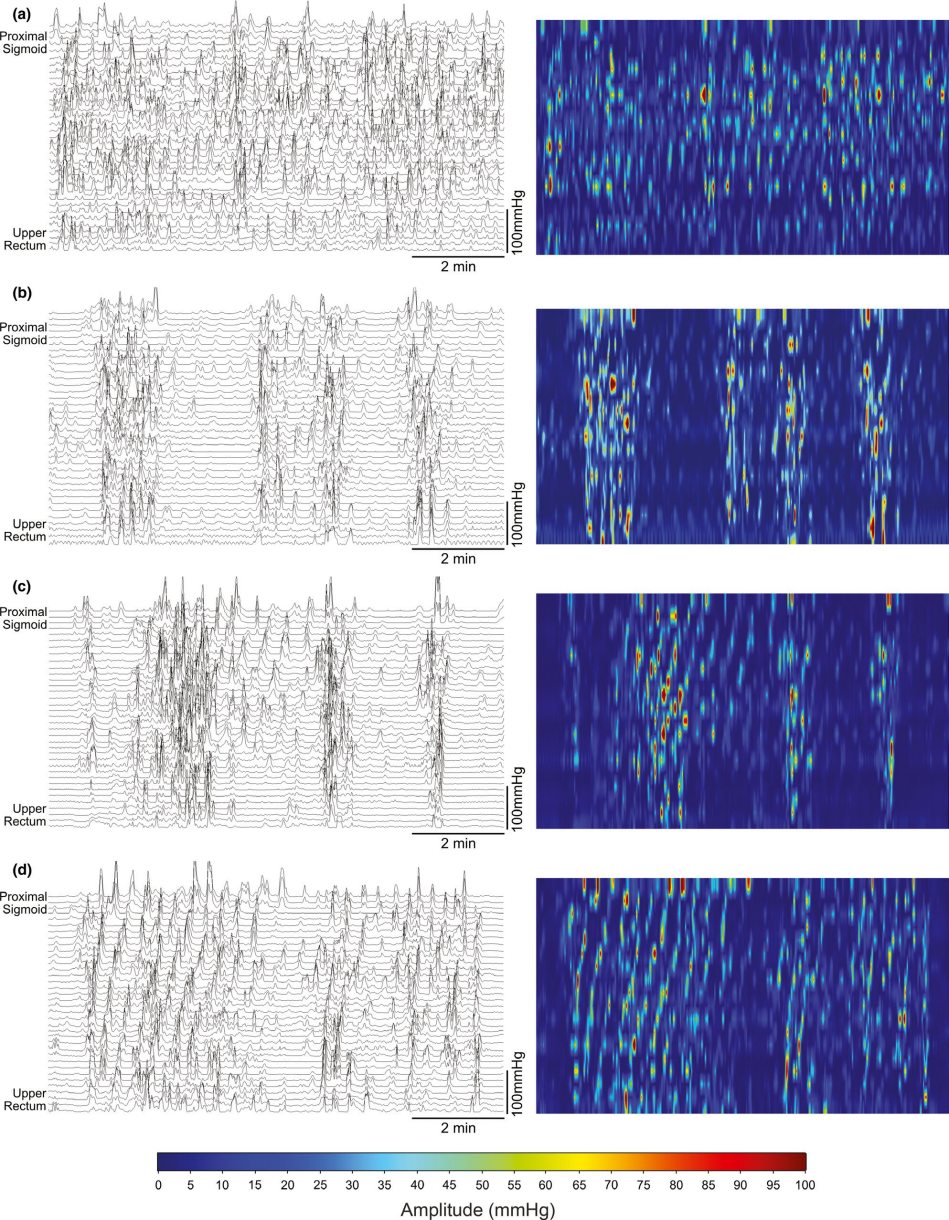
Selected data from 10 min windows, shown as both pressure traces (left) and color plots (right), demonstrating (a) disorganized activity with periods of 2–6 cpm activity occurring out of phase between adjacent regions, (b and c) alternating periods of hyperactivity and quiescence with both 2–6 cpm and 8–12 cpm activity, and (d) short period of hyperactivity resembling propagating retrograde cyclic motor patterns, observed during the 14–16 h period

**FIGURE 3:**
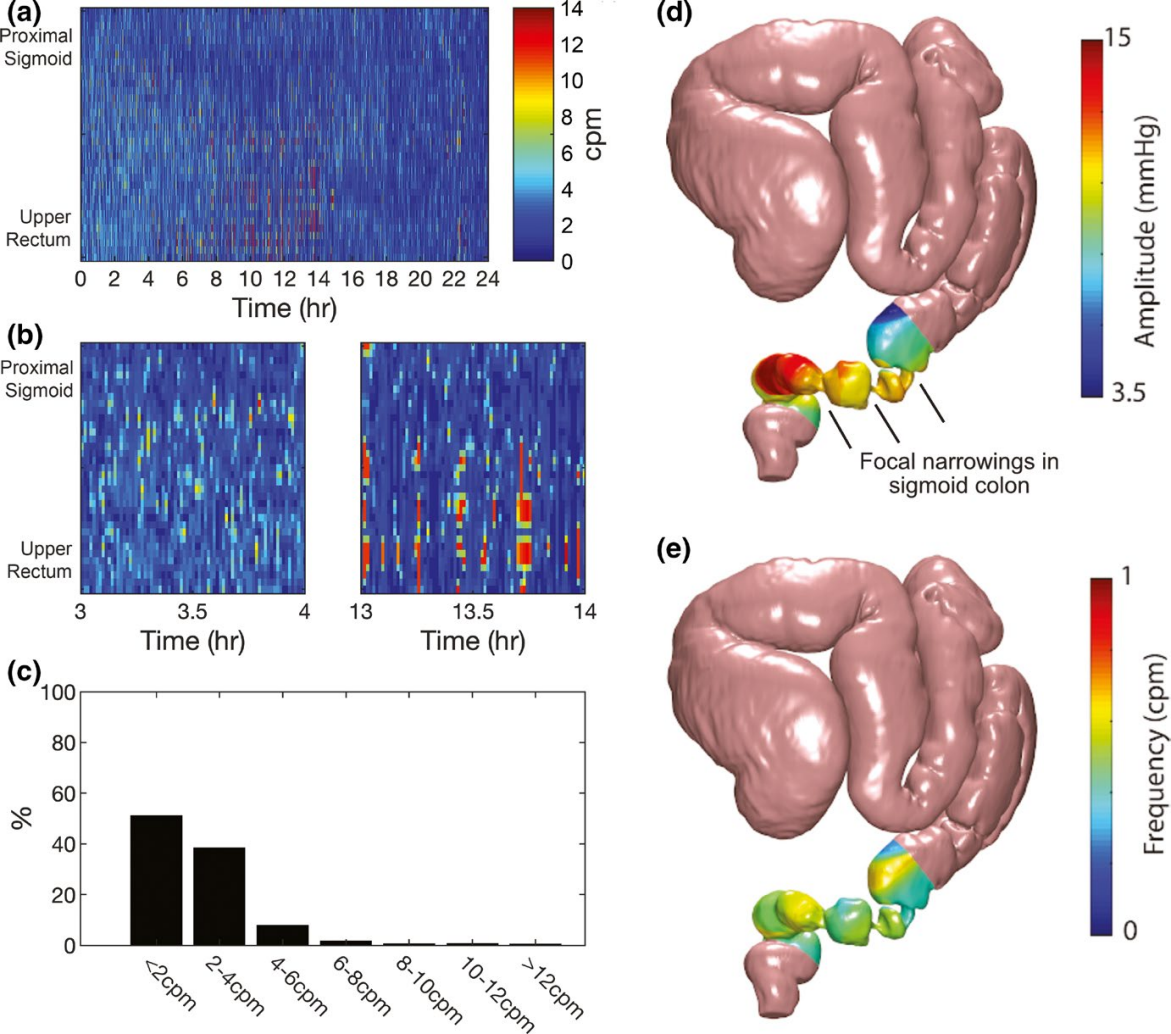
(a) Frequency plot between the 0 and 4 cpm range over the 24 h recording period. (b) Frequency plot between the 0 and 1 cpm range over the 24 h recording period. (c) Percentage of recording occupied by signals with <1 cpm, 1–2 cpm, 2–3 cpm, and >4 cpm frequencies. (d) Mean amplitude and dominant frequency (e) over the 24 h recording mapped onto the three‐dimensional model. Focal narrowings in the sigmoid colon have been marked

The distal colon was profoundly hyperactive during the 24 h recording (Figure 1b). Motor activity in the first 4–6 h of recording was hyperactive, nearly constantly present, and often discontiguous across adjacent regions, with few periods of quiescence (Figure 2a). Due to the relatively disorganized nature of the motor activity and large number of pressure events occurring, it was not possible to classify motor patterns according to the previously published consensus terminology or patterns observed in healthy controls (Corsetti et al., 2019; Dinning et al., 2014). Cyclic activity at 2–6 cpm was common and was often near‐synchronous, though some waves appeared to propagate for short distances in antegrade and retrograde directions (Figure 2). This activity occurred over short distances of <5 cm (Figure 2a), was not organised into propagating cyclic motor patterns (CMP), and was often out‐of‐phase with activity occurring in adjacent regions of the colon (Figure 2a). FFT analysis confirmed that 2–6 cpm activity predominated in the first 6 h (Figure 3a). The 3D reconstruction of the pseudo‐obstructed colon demonstrated several points of focal narrowing in the sigmoid colon within this same hyperactive region (Figure 3d, Video S2).

During the 8–14 h (nighttime) period, there were alternating periods of hyperactivity and quiescence occurring across all sensors (Figure 2b,c). During this time, hyperactivity often lasted for 1–2 min, with contractions of up to 100 mmHg, and occurred irregularly, interspersed by periods of relatively low activity lasting for 1–3 min (Figure 2c). Both 2–6 cpm activity and a higher frequency range of 8–12 cpm were observed during these contractions (Figures 2b,c; 3a,b). Occasional short (<10 min) periods of activity resembling CMP were observed during the 14–16 h recordings (Figure 2D).

The mean amplitude across all sensors was relatively consistent for the first 16 h (mean amplitude 11.3 mm Hg, standard deviation 1.9 mm Hg) (Figure S1), and higher than previously reported pre‐ (~2 mm Hg) and post‐prandial (~4–6 mm Hg) data from healthy controls (Dinning et al., 2014).

## 4 DISCUSSION

This is the first report of colonic motility patterns in ACPO and has demonstrated a novel mechanism for the underlying functional large bowel obstruction. HR colonic manometry demonstrated that the distal colon was dominated by hyperactive, non‐propagating motor activity that was relatively disorganized compared to the previous descriptions of CMP (Lin et al., 2017; Vather et al., 2018). Propagating patterns such as CMP were infrequently seen and high‐amplitude propagating sequences (HAPS) were not observed. The 3D colonic model generated from CT imaging was concordant with the manometry, demonstrating regions of focal marked narrowing that likely corresponded to the recorded strong colonic contractions. It is interesting to note that transient “ring[s] of spasm” in the distal colon was noted during laparotomy in several early descriptions of ACPO (Macfarlane & Kay, 1949; Ogilvie, 1948), which would correlate with the high‐amplitude contractions seen in HR manometry recordings and the regions of narrowing visualized on the 3D model. The distal colonic hyperactivity in this patient was relatively non‐propagating and disorganized compared to previously reported postprandial and postoperative motility patterns (Lin et al., 2017; Vather et al., 2018). These patterns would likely be non‐propulsive, resulting in a functionally obstructed distal colon, leading to the pseudo‐obstruction and proximal colonic dilatation.

In healthy individuals, a 'rectosigmoid brake' motor pattern is observed in this region of the gut postprandially, and is characterised by CMP occurring at 2‐6 cpm, thought to be patterned by neural activity in concert with myogenic mechanisms (Lin et al., 2017). Hyperactive CMP also occur in the distal colon following right hemicolectomy and may contribute to delayed gut transit in postoperative ileus (Seo et al., 2021; Vather et al., 2018). It has been hypothesized that this hyperactivity results from sympathetic activation and neurohormonal changes in the surgical stress response (Vather et al., 2018). While 2–6 cpm hyperactivity was present in this patient with ACPO, this was considerably less organized than the CMP observed postprandially or postoperatively (Lin et al., 2017; Vather et al., 2018), and was mostly non‐propagating, suggesting that other abnormalities affecting the propagation of coordinated contractile activity such as gap junction dysfunction may play a role. Whether this 2–6 cpm cyclic activity arises from haustral boundaries (Huizinga et al., 2021), other dominant pacemaker regions (Lin et al., 2017), or via other mechanisms remains an area requiring further investigation. The sphincter of O'Beirne has recently been characterized with HR manometry as a 1 cm high‐pressure region at the rectosigmoid junction and its overactivity is hypothesized to contribute to some cases of chronic constipation (Chen, Collins, et al., 2021; Chen et al., 2021), though the hyperactivity observed in this patient with ACPO was present across a much longer segment of colon. High‐frequency activity at ~12 cpm has previously been demonstrated in recordings from the human colon (Pervez et al., 2020; Sarna et al., 1981), and short periods of activity at this frequency were also observed.

Inflammatory, pharmacological, and cellular factors are thought to contribute to ACPO, though excessive sympathetic drive and/or reduced parasympathetic activity appear to be a common pathophysiological pathway (Wells et al., 2017). Overactive noradrenergic signaling may suppress neurogenic HAPS, and unmask intrinsic pacemaker activity in the distal colon (Hanman et al., 2019; Rosli et al., 2020). No HAPS were observed in 24 h of manometric recordings from this patient, although events may have been missed due to the distal location of the manometry catheter. It is interesting to note that this patient had a prior episode of ACPO, as well as a previous severe episode of *C*. *difficile* colitis. It is unclear whether these or other unknown factors predisposed her to disordered colonic motility during periods of excessive sympathetic tone.

The disorganized distal colonic hyperactivity observed in this patient may act as a functional obstruction to colonic transit, resulting in proximal colonic distension. However, proximal colonic distension may also have effects on motility. Experimental studies with short periods (5 min) of balloon distension in the proximal colon have elicited HAPS, simultaneous pressure waves, and CMP at both 2–6 cpm and 11–13 cpm (Chen et al., 2018; Pervez et al., 2020); however, it is unclear how this translates to the setting of ACPO. Colo‐colonic reflex arcs have been suggested as a potential mechanism of ACPO (Wells et al., 2017); however, the physiological effects of sustained severe distension on distal colonic motility remain uncertain. Given the recordings in this study were obtained following endoscopic decompression, it is unlikely that colonic distension would explain the sustained dysmotility observed over the 24 h period.

Neostigmine is an effective treatment for colonic decompression in ACPO, though was not used in this patient due to her history of cardiac disease. Acetylcholinesterase inhibition and increased cholinergic signaling in the colon trigger HAPS (Law et al., 2001), which could overcome the distal colonic hyperactivity and functional obstruction. However, this hypothesis remains speculative and the precise effects of neostigmine on abnormal colonic motility patterns in ACPO should be further investigated with HR manometry in future studies.

Given the vast array of pathologies associated with ACPO (Nanni et al., 1982), it is unclear whether all cases are due to the mechanism observed in this patient and there may be heterogeneous dysmotility patterns resulting in ACPO (Wells et al., 2017). Despite considerable recruitment effort over more than 2 years to corroborate this novel report, we have been unable to obtain further data from additional subjects due to the sporadic and acute nature of ACPO, the systemic illnesses of many patients, and the invasive recording techniques used. This analysis is limited by the single case and the lack of baseline recordings prior to decompression or during a non‐obstructed period; however, to our knowledge, it is the only colonic motility data ever reported from a patient with ACPO. The noise removal methods used may have removed pancolonic events, though this approach was used to avoid potential bias from movement or cough artifacts. Finally, it is unclear whether these disorganized motility patterns will be able to be detected by emerging techniques such as non‐invasive body surface mapping (Erickson et al., 2020), and further investigation may require HR manometry or other invasive methods. Further work is needed to confirm the findings of this case report, which present a potential novel mechanism of disease and potential target for future therapies in ACPO.

## CONFLICTS OF INTEREST

GOG, PD, and AG are shareholders and members of Alimetry and hold intellectual property in the field of non‐invasive gastric mapping. PD and NP hold intellectual property in the field of gastric electrophysiology and are shareholders in FlexiMap Ltd. No commercial financial support was received for this study. The other authors have no conflicts of interest to declare.

## AUTHOR CONTRIBUTIONS

CW, IB, and GOG conceived the study. All authors were involved in the acquisition, analysis or interpretation of data for the work and drafting the work or revising it critically for important intellectual content. All authors approved the final version of the manuscript and agreed to be accountable for all aspects of the work in ensuring that questions related to the accuracy or integrity of any part of the work are appropriately investigated and resolved.

## Supporting information



Fig S1

Fig S2

Video S1

Video S2
