# Supplementary figures and images for: A novel mechanism for acute colonic pseudo‐obstruction revealed by high‐resolution manometry: A case report
Source: Physiol Rep. 2021 Jul 7;9(13):e14950. doi: 10.14814/phy2.14950 (PMC8261480; doi:10.14814/phy2.14950)

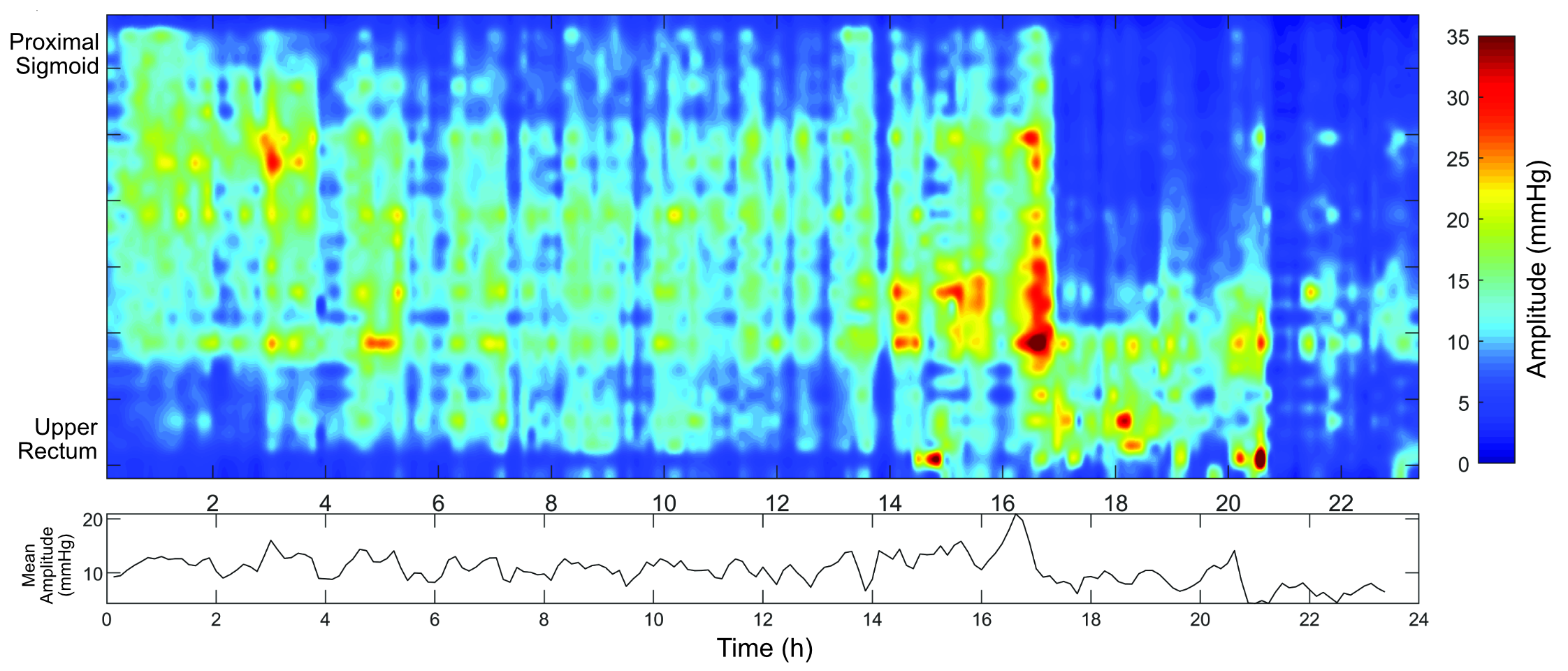

Supplement: Supplementary file 1 — Fig S1 [file PHY2-9-e14950-s001.tif]

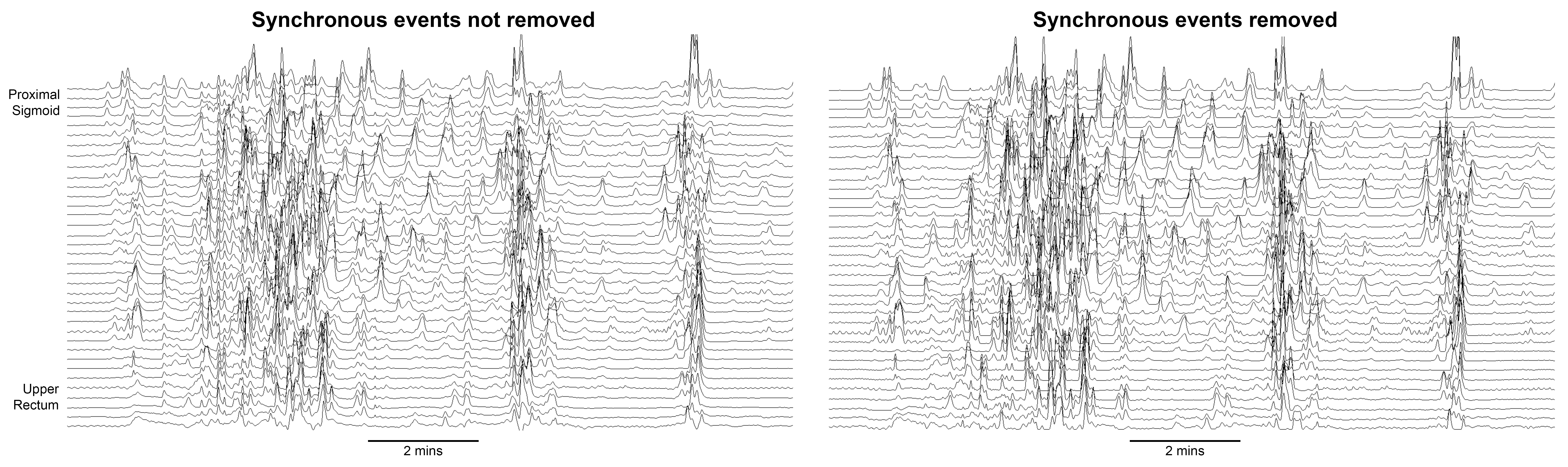

Supplement: Supplementary file 2 — Fig S2 [file PHY2-9-e14950-s004.tif]
